# Risk estimates for ionizing radiation

**DOI:** 10.1590/0100-3984.2023.56.3e5-en

**Published:** 2023

**Authors:** Renato Dimenstein

**Affiliations:** 1 Medical physicist, specialist certified by the Associação Brasileira de Física Médica, radiation safety supervisor at the Comissão Nacional de Energia Nuclear. Email: renato.dimenstein@gmail.com.

According to the literature, cumulative (stochastic) effects of radiation exposure, including cancer and hereditary effects, are caused by a mutation or other permanent changes in which the cell remains viable. Although the severity of the response observed in the stochastic effect does not depend on the minimum threshold, the probability of a stochastic effect increases in parallel with an increase in the radiation dose^(1)^.

Despite the fact that the use of radiation has proven safe for medical purposes, health professionals still have many questions about the risks of exposure, especially those related to nuclear medicine patients. To answer those questions about radiological safety, Willegaignon et al.^(2)^ conducted a study, published in a recent issue of Radiologia Brasileira, on radiation safety measures in diagnostic nuclear medicine. Their simple yet robust methodology allowed the quantification of radiation exposure of patients injected with radioisotopes. The experimental work employed a precalibrated Geiger-Müller detector to estimate patient radiation dose rates. An external dosimetry study evaluated, separately, exposure to different radioisotope energies, levels of radioactivity, radiopharmaceuticals, and clearance times. Measurements with the radiation detector estimated the dose rate (in µSv/h) of individuals occupationally exposed in settings of radioisotope administration, image acquisition, and patient release from the nuclear medicine unit. On the basis of the data collected, the authors estimated the radiation exposure of the patient after leaving the radioisotope sector, as a function of time. What is important in their article is the discrimination of radiation dose values, which showed that individuals in the general population and hospital workers received equivalent dose values lower than the limits established by international radiation safety standards^(3)^.

The methodological limitations of the external dosimetry of the study in question are related to the response times of the detector systems and the Geiger calibration curves for the different radioisotope energies. Despite the uncertainties, the results and conclusion of the Willegaignon et al.^(2)^ study do not change. Therefore, the relevance of their work lies in ensuring the safe use of radioisotopes for occupationally exposed individuals and individuals in the general population, in accordance with radiation safety guidelines^(4,5)^.

The Willegaignon et al.^(2)^ article allows us to conclude that the equivalent doses for individuals in the general population do not exceed the limit of 1 mSv/year established for such individuals^(6)^. In most cases, dose limits are incomprehensible to the public. Therefore, to facilitate communication between radiologists/nuclear physicians and their patients, family members, and other health professionals, it is suggested that radiation levels be characterized in terms of the environmental dose. For example, the annual exposure received by the population, resulting from cosmic rays, is approximately 2.3 mSv year, which is equivalent to the effective dose value of a cranial computed tomography examination. Another example that might be useful is that the dose received by a passenger on a flight between Tokyo and New York, is 0.23 mSv, which does not increase the risk of stochastic effects^(7)^.
